# Evaluation of second-trimester maternal serum betatrophin levels and lipid and carbohydrate metabolism parameters in patients with gestational diabetes mellitus

**DOI:** 10.4274/tjod.galenos.2020.67026

**Published:** 2020-04-06

**Authors:** Funda Gülcü Bulmuş, Rauf Melekoğlu, Mehmet Ferit Gürsu, Helin Bağcı, Ebru Celik Kavak, Alpaslan Akyol

**Affiliations:** 1 Fırat University Faculty of Medicine, Vocational School of Health Services, Elazığ, Turkey; 2 İnönü University Faculty of Medicine, Department of Obstetrics and Gynecology, Malatya, Turkey; 3 Fırat University Faculty of Medicine, Department of Biochemistry, Elazığ, Turkey; 4 Karabük University Training and Research Hospital, Clinic of Obstetrics and Gynecology, Karabük, Turkey; 5 Fırat University Faculty of Medicine, Department of Obstetrics and Gynecology, Elazığ, Turkey; 6 Fırat University Faculty of Medicine, Department of Obstetrics and Gynecology, Elazığ, Turkey

**Keywords:** Betatrophin, gestational diabetes mellitus, insulin resistance

## Abstract

**Objective:**

We investigated the role of betatrophin in the etiopathogenesis of gestational diabetes mellitus (GDM) and its association with lipid and carbohydrate metabolism in patients with GDM and normoglycemic pregnant women.

**Materials and Methods:**

A total of 60 patients [30 pregnant women with GDM (study group) and 30 healthy age-, body mass index-, and gestational agematched pregnant women (control group)] were included in this study. Serum betatrophin, fasting glucose, insulin, glycated hemoglobin A1c (HbA1c), and C-peptide levels, as well as lipid parameters, were measured.

**Results:**

Serum betatrophin, fasting glucose, HbA1c, insulin, and C-peptide levels were significantly higher in the GDM group than in the control group (p<0.001, p=0.009, p=0.013, p<0.001, and p<0.001, respectively). Levels of triglycerides and very-low-density lipoprotein cholesterol were significantly higher in the GDM group (p=0.020 and p=0.020, respectively), but total cholesterol and LDL cholesterol levels were similar in the two groups (p=0.810 and p=0.273, respectively). Betatrophin levels in the GDM group were correlated positively with insulin levels (r=0.336, p=0.009) and the homeostatic model assessment of insulin resistance (HOMA-IR) score (r=0.269, p=0.038), and negatively with the C-peptide levels (r=-0.399, p=0.002); they were not correlated with any other glucose or lipid parameters. Multivariate stepwise linear regression analysis demonstrated that insulin levels (β=0.134, p=0.013) and the HOMA-IR score (β=0.112, p=0.017) were associated independently with serum betatrophin levels.

**Conclusion:**

These results demonstrate that serum betatrophin levels were significantly higher in pregnant women with GDM than in normoglycemic pregnant women. The levels of betatrophin were correlated significantly with insulin resistance parameters, which is a key feature of GDM pathophysiology.

**PRECIS:** In this study, we compared serum levels of betatrophin in women with and without gestational diabetes mellitus, and investigated the relationships between betatrophin and lipid and glucose metabolism parameters.

## Introduction

Gestational diabetes mellitus (GDM) is defined as impaired carbohydrate tolerance characterized by severe hyperglycaemia that is recognized for the first time during pregnancy^(1)^. GDM affects approximately 7% of all pregnancies; the rate varies from 1-28% depending on the diagnostic criteria and the study population^(2)^. GDM is associated with increased maternal, foetal, and neonatal risks, including preterm birth, hypertensive disease of pregnancy, macrosomia, polyhydramnios, foetal death, operative delivery, caesarean delivery, birth trauma, hypoglycaemia, hyperbilirubinemia, and respiratory distress syndrome^(3)^. GDM treatment is associated with significant reductions in primary outcomes from severe complications, such as perinatal death, shoulder dystocia, birth trauma, macrosomia, and preeclampsia^(4)^. Thus, screening for GDM is recommended for all pregnant women at 24-28 weeks’ gestation, using a laboratory-based screening test and blood glucose levels when the diabetogenic effects of pregnancy are evident^(5)^.

Betatrophin is a recently identified circulating endocrine hormone that is secreted primarily by the liver and adipose tissue and plays an essential role in glucose homeostasis by promoting beta-cell proliferation^(6)^. This hormone, also known as lipasin, hepatocellular carcinoma-associated protein TD26, angiopoietin-like protein 8, and refeeding-induced fat and liver protein, also plays an important role in lipid metabolism by inhibiting lipoprotein lipase and reducing triglyceride clearance^(7)^. Studies have demonstrated that the expression of betatrophin is induced by insulin, food intake, and cold exposure, but is suppressed by starvation^(8,9)^. Some researchers noted that overexpression of betatrophin improved glucose tolerance by promoting β-cell proliferation and insulin production, whereas others failed to find any association between the expression of betatrophin and β-cell growth^(10,11)^. Increasing evidence suggests an association between altered betatrophin levels and type 2 DM or obesity, but the correlation between betatrophin expression and GDM is controversial. In addition, the effect of the betatrophin level on glucose and lipid metabolism has been controversial in several studies. Therefore, in this study, we compared serum levels of betatrophin in women with and without GDM, and investigated the relationships between betatrophin and lipid and glucose metabolism.

## Materials and Methods

This study was approved by the Local Ethics Committee of Fırat University, and informed consent was obtained from all participants in accordance with the principles of the Declaration of Helsinki (approval no: 97132852/050.01.04). Sixty participants [30 pregnant women with GDM (study group) and 30 healthy age-, gestational age- and body mass index (BMI)-matched pregnant women (control group)] were enrolled in this prospective case-control study from the Fırat University Faculty of Medicine, Department of Obstetrics and Gynaecology between January 2017 and January 2018.

The inclusion criteria were maternal age 18-39 years, viable singleton pregnancy, admission for GDM screening at 24-28 weeks’ gestation, BMI <35 kg/m^2^, and an unremarkable medical or obstetric history. Exclusion criteria were the presence of any congenital malformation or chromosomal abnormality, foetal death, multiple pregnancies, maternal polycystic ovary syndrome, pregestational DM, family history of DM in a first-degree relative, hypertensive disease during pregnancy, chronic maternal disease (chronic hypertension, dyslipidaemia, chronic renal failure, pulmonary or cardiac disease, and malignancy), the use of any medication that interferes with lipid or glucose metabolism, and smoking or alcohol consumption. Gestational age was determined from the first day of the last menstrual period and confirmed by first trimester or early second-trimester ultrasonography.

GDM screening was performed in all participants between 24 and 28 gestational weeks using the 75-g oral glucose tolerance test (OGTT), as defined by the International Association of Diabetes and Pregnancy Study group one-step diagnostic approach^(12)^. After fasting for 8-10 h, the patients were requested to drink 75 g anhydrous glucose dissolved in 300 mL water within 5 min, followed by a measurement of venous plasma glucose concentrations 1 and 2 h after ingestion. The diagnosis of GDM was based on a single serum glucose level that met or exceeded the cut-off values (fasting glucose, 92 mg/dL; 1 h value, 180 mg/dL; 2 h value, 153 mg/dL). Blood samples were centrifuged at 3500 rpm for 5 min to separate sera. Fasting plasma glucose levels and fasting serum levels of insulin and C-peptide were used to evaluate pancreatic β-cell function; we also measured serum levels of lipid metabolism parameters [total cholesterol; low-density-lipoprotein (LDL), high-density-lipoprotein (HDL), and very-low-density-lipoprotein (VLDL) cholesterol; and triglycerides]. Betatrophin levels and other biochemical parameters were measured on the day of OGTT screening. Glucose, lipid, and glycated hemoglobin A1c (HbA1c) levels were analyzed on an Olympus AU 2700 autoanalyzer (Olympus Optical Co., Tokyo, Japan). The homeostatic model assessment of insulin resistance (HOMA-IR) score, calculated by the formula defined by Matthews et al.^(13)^, was used to measure insulin resistance [fasting insulin level (µU/mL) ´ fasting glucose level (mmol/L)/22.5]. BMI was measured during OGTT screening using the calculation: weight (kg)/height (m)^2^.

Serum levels of betatrophin were determined using an enzyme-linked immunosorbent assay (ELISA; catalogue no. E-EL-H2206; Elabscience, Houston, TX, USA). Absorbance at 450 nm was recorded using an ELX800 ELISA reader. An automated model ELX50 washer was used to wash the plates. The assay results are expressed in pg/mL (range: 125-800 pg/mL).

### Statistical Analysis

A power analysis (PASS 11; NCSS, LLC, Kaysville, UT, USA; www.ncss.com) suggested that at least 29 subjects should be included in each group if the greatest between-group difference in the betatrophin level was 11.12 ng/mL, with a standard deviation (SD) of 4.3 ng/mL, type I error of 0.05, and type II error of 0.10^(14)^. We recorded age, gravidity, parity, BMI, gestational age at screening, and perinatal outcomes of the patient and control groups. The Statistical Package for the Social Sciences version 22.0 software (SPSS Inc., Chicago, IL, USA) was used for the data analysis. Data are reported as means ± SDs, medians (ranges), or percentages. The normality of data distribution was checked using the Shapiro-Wilk test. Student’s t-test was employed to compare biochemical parameters between the control and study groups. The Mann-Whitney U test was used to compare data when dependent variables were not normally distributed. The chi-square test and Fisher’s exact test were used to compare categorical variables between the groups, as appropriate. Univariate correlations were analyzed using a non-parametric Spearman’s correlation test. Multivariate stepwise linear regression was used to detect independent relationships between the metabolic parameters and serum betatrophin levels. p values <0.05 were considered to be significant.

## Results

The maternal characteristics and perinatal outcomes of the study and control groups are summarized in Table 1. Patients were matched in terms of age, BMI, and gestational age at screening, and these parameters did not differ significantly between the groups. Gravidity, parity, and perinatal outcomes were also similar in the two groups.

As expected, serum fasting glucose, insulin, and HbA1c levels, as well as HOMA-IR scores, were significantly higher in pregnant women with GDM than in controls (p=0.009, p<0.001, p=0.013, and p<0.001, respectively). Total cholesterol and LDL cholesterol levels were similar in the two groups (p=0.810 and p=0.273, respectively). VLDL cholesterol and triglyceride levels were significantly higher in the GDM group than in the control group (both p=0.020). Glucose and lipid metabolism parameters are summarized in Table 2.

**Figure 1:**
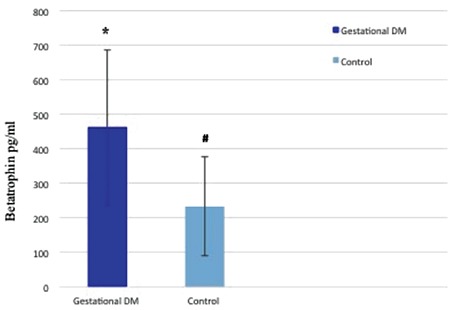
Differences in betatrophin levels between groups. Values are means ± standard deviations (pg/mL). * and # indicate significant differences (p<0.001). Gestational diabetes mellitus: 463.19±224.64, control: 233.13±143.63, p<0.001. DM: Diabetes mellitus

Serum betatrophin levels were significantly higher in patients with GDM than in the control group (p<0.001; Figure 1). No significant correlation was evident between betatrophin levels and glucose or lipid metabolism parameters in the control group. However, significant correlations were detected between the betatrophin levels and insulin levels (r=0.336, p=0.009), C-peptide levels (r=-0.399, p=0.002), and HOMA-IR scores (r=0.269, p=0.038) in the GDM group; they were not correlated with any other glucose or lipid parameter. The results of correlation analyses for the GDM and control groups are summarized in Table 3. Multivariate stepwise linear regression analysis revealed that the insulin levels (β=0.134, p=0.013) and HOMA-IR values (β=0.112, p=0.017) were independently related factors associated with serum betatrophin levels.

## Discussion

The data presented here demonstrate that serum circulating betatrophin levels were significantly higher among pregnant women with GDM than among normoglycemic (control) pregnant women. In recent years, changes in various hepatocyte- and adipocyte-derived factors, such as adiponectin, resistin, leptin, and adipocyte fatty acid-binding protein, have been reported as mediators for the regulation of pregestational DM and GDM^(15)^. In addition, betatrophin has been identified as a new adipokine/hepatokine, and has been claimed to have important roles in promoting β-cell proliferation and β-cell mass expansion in animal and human studies^(16,17)^. Sun et al.^(18)^ demonstrated that the transplantation of betatrophin-expressing adipose-derived mesenchymal stem cells induced β-cell proliferation in mice with streptozotocin (STZ)-induced diabetes^(18)^. They showed that betatrophin overexpression induced pancreatic islet proliferation, β-cell-specific transcription factor expression, and insulin production by islet cells under glucose stimulation. In contrast, Gusarova et al.^(19)^ failed to show growth of beta cells in mice in response to targeted ANGPTL8 overexpression. Jiao et al.^(20)^ demonstrated that betatrophin stimulated β-cell replication in an experimental S961-induced insulin resistance model in mice, but they did not increase human β-cell DNA replication in the transplanted setting^(20)^. These inconsistent results of increased betatrophin may be due to the experimental models used (i.e., STZ-induced diabetes vs. S961-induced insulin resistance). In particular, the S961-induced insulin resistance model may be effective for shorter-duration experiments. Moreover, the proliferative potential of pancreatic islet cells can vary among mouse age groups, which may have biased the outcomes. Although the mechanism underlying the effect of betatrophin on glucose homeostasis has been attributed to increased β-cell proliferation, the precise impact of betatrophin on insulin secretion remains unclear, and further studies are needed to elucidate the exact mechanism.

In view of the frequency of GDM, several animal and human studies have been conducted to identify potential pathophysiological factors for insulin resistance in women with GDM. As a novel biomarker of glucose and lipid metabolism, betatrophin has been suggested to have a regulatory role in insulin resistance in patients with GDM^(21)^. The results of the present study also reveal that insulin levels and HOMA-IR scores are associated independently with serum betatrophin levels. Chen et al.^(22)^ reported a significant increase in the betatrophin level in patients with type 2 DM, and showed significant correlations between the betatrophin level and insulin resistance indices, including the HOMA-IR score. Kong et al.^(23)^ conducted a meta-analysis to evaluate the association between circulating betatrophin levels and GDM, and indicated that circulating betatrophin was evident in patients with GDM, especially in those with BMIs ≥28 kg/m^2^ during the third trimester. They proposed that a higher tendency for insulin resistance in the third trimester of pregnancy and in obese patients may have contributed to these results, obtained from a subgroup analysis. Wawrusiewicz-Kurylonek et al.^(24)^ showed increased maternal circulating betatrophin levels in patients with GDM, and the levels were about five times higher in cord blood than in maternal serum. They noted a negative correlation between maternal betatrophin and serum C-peptide concentrations, and suggested that decreased insulin secretion capability altered the betatrophin level.

Lipid metabolism significantly changes through pregnancy, due mainly to an increase in adipose tissue followed by increased lipolysis and hypercholesterolemia^(25)^. Changes in oestrogen and progesterone have been proposed to contribute significantly to this physiologic hyperlipidaemia. However, the association between increased adiposity and circulating betatrophin in maternal serum has not been clearly defined^(26)^. In this study, we observed no correlation between the serum betatrophin levels and lipid parameters in the GDM or control group. Similarly, Erol et al.^(27)^ evaluated circulating betatrophin levels and metabolic parameters in women with GDM and found no correlation between betatrophin levels and lipid parameters, including triglyceride, total cholesterol, LDL cholesterol, and HDL cholesterol levels. By contrast, Fenzl et al.^(28)^ demonstrated a significant association between betatrophin levels and atherogenic lipid profiles in patients with morbid obesity or type 2 DM. These conflicting results may be attributed to differences in study design, sample size, or the immunoassay kits used for laboratory assessment. Further studies with larger samples are needed before more definitive conclusions can be made.

### Study Limitations

The current study has several limitations, such as the examination of a small sample from a single centre. However, the number of patients participating in the study was sufficient to analyse the serum betatrophin level and metabolic parameters. The change in betatrophin during the course of pregnancy was not determined because we were only able to analyse second-trimester serum betatrophin levels. The main strength of the study was the prospective cohort design with strict inclusion and exclusion criteria, which minimised the effects of potential confounding factors. A further strength was that the study and control groups were well matched in terms of baseline characteristics.

## Conclusion

This study demonstrated that serum betatrophin levels were significantly higher in pregnant women with GDM than in normoglycemic pregnant women. The level of betatrophin was correlated significantly with insulin resistance parameters, which is a key feature of GDM pathophysiology. These findings may help to clarify the pathophysiology of GDM and may be useful for the prediction of GDM development during the second trimester.

## Figures and Tables

**Table 1:**
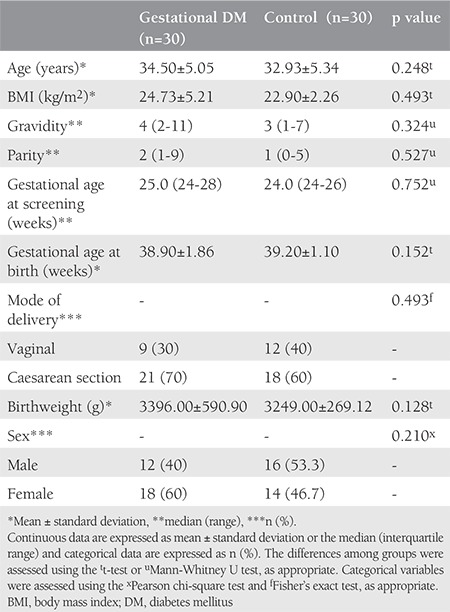
Maternal characteristics and birth outcomes of the study and control groups

**Table 2:**
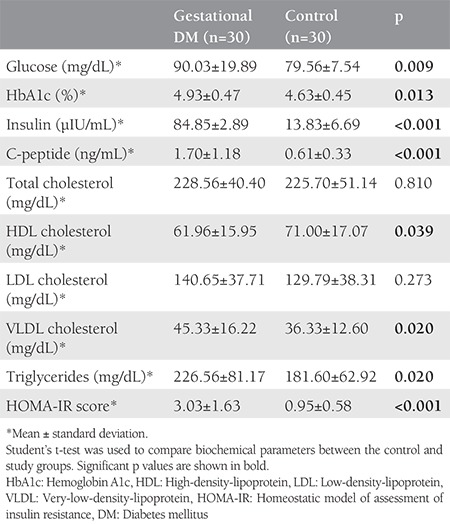
Carbohydrate and lipid metabolism parameter levels

**Table 3:**
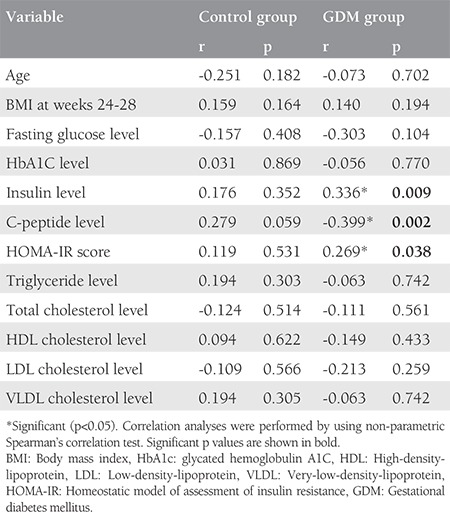
Correlations between the betatrophin level and clinical and biochemical parameters in the GDM and control groups in weeks 24-28 of pregnancy
